# Health-related quality of life with adjuvant immune checkpoint inhibition in renal cell carcinoma: A qualitative study of UK participants in the phase III RAMPART trial

**DOI:** 10.1371/journal.pone.0358789

**Published:** 2026-09-21

**Authors:** Sophie Merrick, Annabelle South, Elena Frangou, Hannah L. Rush, Grisma Patel, Jemima Thompson, Hannah Plant, Lisa Pickering, Ruth E. Langley, Duncan C. Gilbert, Thomas Powles, James Larkin, Angela Meade

**Affiliations:** 1 UCL Innovative Clinical Trials Unit, Institute of Clinical Trials and Methodology, UCL, London, United Kingdom; 2 Guy’s and St Thomas’ NHS Foundation Trust, London, United Kingdom; 3 The Royal Marsden Hospital NHS Foundation Trust, London, United Kingdom; 4 St Bartholomew’s Hospital, London, United Kingdom; Mayo Clinic Cancer Center, UNITED STATES OF AMERICA

## Abstract

**Introduction:**

RAMPART is an international phase III trial evaluating adjuvant immune checkpoint inhibitors (ICIs) after nephrectomy for renal cell carcinoma. ICIs have a distinct toxicity profile that can significantly impact health-related quality of life (HRQoL); however existing HRQoL questionnaires were developed in a pre-ICI era and may not fully capture the patient experience. To address this, we embedded a qualitative interview sub-study within RAMPART among UK participants to explore the impact of ICIs on HRQoL in depth.

**Methods:**

Eligible participants were enrolled in RAMPART at UK sites and randomised to Arm B (durvalumab q4w for 1 year) or C (durvalumab q4w for 1 year plus tremelimumab at day 1 and week 4). Participants were purposively selected to ensure representation across baseline characteristics, treatment tolerance, toxicity development, and recurrence status. Interviews were conducted online, via telephone and in person, with transcripts analysed using the framework method.

**Results:**

Of 220 eligible participants, 71 were invited; with recruitment closing early due to a high response rate. 37 expressed interest, and 23 were interviewed. Two themes were generated based on participant insights: *Living in a Changed Body and Mind* and *It Depends on Your Life: How Context Shapes the Impact of Immune Related Adverse Events (irAEs)*. The findings suggest that participants often placed greater importance on the duration and chronicity of irAEs than their severity when describing impacts on HRQoL. Persistent toxicities, including fatigue, arthralgia, and diarrhoea, disrupted daily functioning, while cognitive changes and emotional strain were common. Employment, financial security, and caregiving responsibilities influenced the impact of irAEs, whereas supportive relationships and structured follow-up mitigated their burden.

**Conclusions:**

Chronic irAEs can negatively affect HRQoL, mental health, and the ability to work. Clinical trials evaluating ICIs should report toxicity grade, duration, and resolution to better inform patient decision-making. Development of ICI-specific HRQoL tools that capture the impact of persistent irAEs is needed, alongside clear care pathways for management of chronic irAEs.

## Introduction

Locoregional renal cell carcinoma (RCC) is primarily managed with radical or partial nephrectomy [1]. However, patients at intermediate or high risk of recurrence face substantial relapse rates, prompting investigation into adjuvant systemic therapies. Immune checkpoint inhibitors (ICIs) have therefore been evaluated in this setting with mixed results. Pembrolizumab has demonstrated improvements in disease free survival (DFS) and overall survival (OS) in patients with resected clear cell RCC [2,3]. Several other perioperative or adjuvant trials have not met their primary endpoints [4–7]. Within this evolving landscape, the RAMPART trial, is evaluating adjuvant durvalumab (anti–programmed death-ligand 1) alone or in combination with tremelimumab (anti–cytotoxic T-lymphocyte–associated protein 4) in patients at intermediate or high risk of relapse. At the primary analysis, durvalumab plus tremelimumab significantly improved DFS compared with active monitoring, while durvalumab monotherapy did not [8].

While DFS and OS are paramount, adjuvant therapy must also be assessed for its impact on health-related quality of life (HRQoL) [9,10]. ICIs present specific challenges: immune-related adverse events (irAEs) can arise unpredictably, affect any organ system, and persist long after treatment cessation [11–17]. Some irAEs necessitate prolonged immunosuppression or lifelong hormone replacement, introducing chronic health burdens for patients who may otherwise be cured [11,13,15]. Understanding how acute and chronic irAEs impact HRQoL is critical to informed decision-making. Regulatory agencies and Health Technology Assessment bodies increasingly require HRQoL outcomes to inform benefit–risk assessment and policy decisions [18].

HRQoL in oncology trials is typically evaluated using patient-reported outcome measures (PROMs). Common instruments include the European Organisation for Research and Treatment of Cancer Quality of Life Questionnaire-Core 30 (EORTC QLQ-C30), the EuroQol 5-Dimension (EQ-5D), and the Functional Assessment of Cancer Therapy (FACT) questionnaires [19,20]. However, these instruments were developed in a pre-immunotherapy era and lack sensitivity to irAEs such as rash, arthralgia, and endocrinopathies as well as prolonged toxicities and the burden of ongoing immunosuppression. The HRQoL impact of many of these chronic or fluctuating effects is still poorly understood. Multiple systematic reviews have highlighted that existing PROMs lack adequate content validity for patients receiving ICIs, raising concerns that they may underestimate treatment burden and fail to capture survivorship issues unique to ICIs [19, 21, 22]. Trial assessment schedules may also fail to identify late onset or persistent irAEs.

To address these limitations in understanding the impact of ICIs on HRQoL, we conducted a qualitative sub-study within RAMPART (NCT03288532) to explore patient experiences beyond the scope of standardised questionnaires. To our knowledge, this is the first qualitative study to examine the impact of adjuvant ICI on HRQoL in RCC. Findings inform clinical decision‑making, trial design, survivorship care and future research. We present the results alongside recommendations co‑developed with patients and multidisciplinary experts.

## Methods

### Study design and research team

This qualitative interview study was embedded within the RAMPART trial and conducted within a critical realist paradigm [23]. This approach recognised participants’ accounts as reflecting real experiences of treatment-related toxicity while also acknowledging that these experiences were shaped by individual contexts. Consequently, analysis considered not only the direct effects of irAEs but also how personal circumstances influenced their impact on HRQoL. Consistent with critical realism, interpretation was understood to be influenced by researchers’ backgrounds and perspectives; therefore, analysis was undertaken collaboratively by clinical and non-clinical researchers to support reflexive interpretation of the data. The research team comprised three oncology clinicians (SM, HLR, GP) and two non-clinical researchers (AS, JT). SM, a female clinical research fellow, conducted all interviews. SM, HLR, AS, and JT had prior qualitative research experience; GP contributed clinical expertise. A patient and public involvement (PPI) representative with lived experience of RCC contributed to the study design, participant materials and interview guide, which was piloted with a patient.

This study is reported in accordance with the Consolidated Criteria for Reporting Qualitative Research (COREQ) checklist [24]. The interview guide and COREQ checklist are provided in the Supporting Information (S1 and S2 File).

### Participants and recruitment

Eligible participants were enrolled in RAMPART at UK sites, received ICI in Arm B (durvalumab 1500 mg every 4 weeks for 1 year) or Arm C (durvalumab 1500 mg every 4 weeks for 1 year plus tremelimumab 75 mg at day 1 and week 4), and had completed ≥2 QLQ-C30 questionnaires (used for eligibility only; questionnaire data not included in this analysis).

Participants were identified centrally and invited by post through research nurses at participating sites. Sites were approached in waves as they activated the sub-study, with later waves targeting underrepresented demographics. Sampling was purposive, aiming for diversity in age, gender, ethnicity, trial arm and range of toxicity experience (see S3 File for purposive sampling framework, including target and achieved recruitment across categories). Recruitment ended once sampling matrix categories were filled or deemed unfillable. Sampling targets were achieved across all predefined characteristics except ethnicity and age ≥ 75 years. Despite targeted recruitment efforts, no participants from Black or Asian ethnic groups expressed interest in participation, and only two participants aged ≥75 years were recruited (target n = 3).

No participants were known to SM (the interviewer) or treated at hospitals where SM worked, and all were aware that SM was an oncology clinician and researcher.

### Sample size

We did not prespecify an exact number of participants. A purposive sampling matrix with minimum targets for age, gender, ethnicity, trial arm and toxicity experience guided recruitment (see S3 File). Sample size was informed by the principle of Information Power, considering the focused study aim, the specificity of the sample, the richness of interview data and use of both within- and cross-case analysis [25]. Recruitment ceased once sampling matrix categories were filled or deemed unfillable and the multidisciplinary research team agreed that the final sample provided sufficient information power to address the study aims [25].

### Ethical approval and consent to participate

Ethical approval was obtained from the London Riverside Research Ethics Committee (17/LO/1875; Substantial Amendment 27, IRAS ID: 219487). All participants provided written informed consent prior to participation. Participants consented to the use of anonymised quotations in study outputs. The study was conducted in accordance with the Declaration of Helsinki and UK General Data Protection Regulation requirements.

Participants were informed that confidentiality would be maintained unless there were concerns regarding risk of harm to themselves or others. A safeguarding procedure was in place whereby any disclosures of significant psychological distress, suicidal ideation, or safety concerns were assessed by the interviewer (SM), a medically qualified oncology clinician, and escalated to appropriate healthcare professionals if required. Participants were also signposted to relevant support services where appropriate. Although some participants described previous experiences of severe depression or suicidal ideation, no active safeguarding concerns requiring escalation were identified during the study.

### Data collection

Baseline characteristics, treatment details, adverse event (AE) data, and concomitant medication were collected prospectively within the RAMPART trial.

Semi-structured interviews were conducted via Microsoft Teams, telephone, or in person, with only the interviewer and participant present. Interviews explored experiences of ICI treatment, irAEs, and perceived impact on HRQoL. Participants were presented with vignettes illustrating severe or chronic irAEs (see S2 File). The interview guide was used flexibly, with participants encouraged to discuss their experiences in their own words. Open-ended questioning and probing were used to explore issues raised by participants in greater depth. Interviews were recorded, transcribed, anonymised, and accompanied by field notes. Participants received a £25 voucher.

### Data analysis

**Fig 1:**
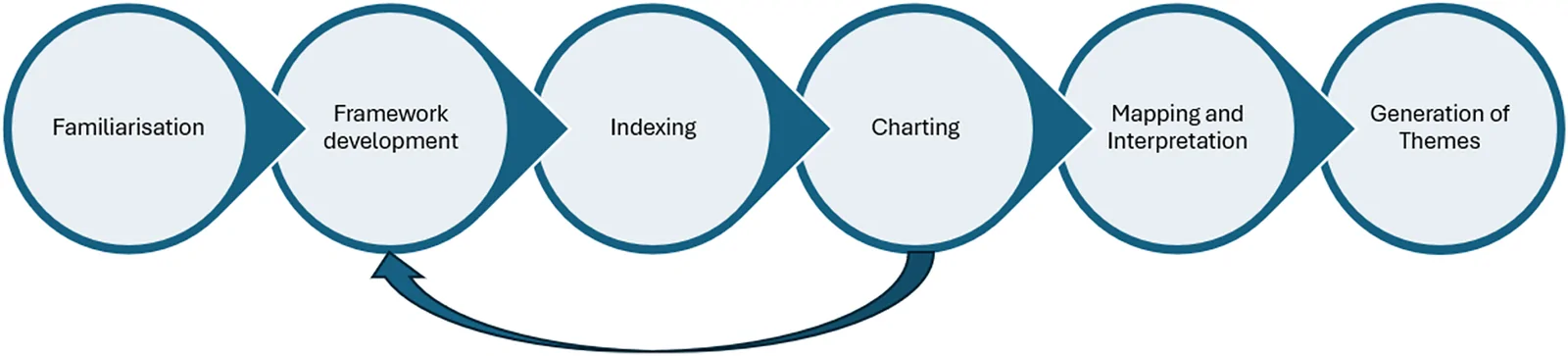
Overview of the framework analysis process. The arrow between framework development and charting indicates that analysis was iterative, with the framework refined as coding and charting progressed.

Transcripts were analysed using the framework method, which involves familiarisation, framework development, indexing, charting, and interpretation [26,27]. These stages were applied iteratively, and an overview is shown in Fig 1 A thematic framework was developed by SM and AS, informed by HRQoL domains and concepts from the data, and refined after initial coding (see S4 File). Transcripts were indexed using this framework, and a matrix of summaries for each domain and case (interviewee) was constructed to facilitate cross-case analysis. Selected excerpts were reviewed in data clinics by AS, HLR, JT, and GP. NVivo14 supported data management [28].

Transcripts were not returned to participants for member checking.

### Recommendation development

Following analysis, preliminary recommendations were drafted based on study findings. We held a PPI workshop with 10 interview participants, where we presented preliminary findings, got their reactions to them, and then asked them to review and refine the draft recommendations. Participants suggested amendments and additions to ensure recommendations reflected patient priorities. The revised recommendations were then reviewed by a multidisciplinary expert panel of clinicians, trialists and methodologists (SM, GP, DG, AM, AS) to confirm clinical relevance and feasibility.

## Results

### Participant recruitment and characteristics

**Fig 2:**
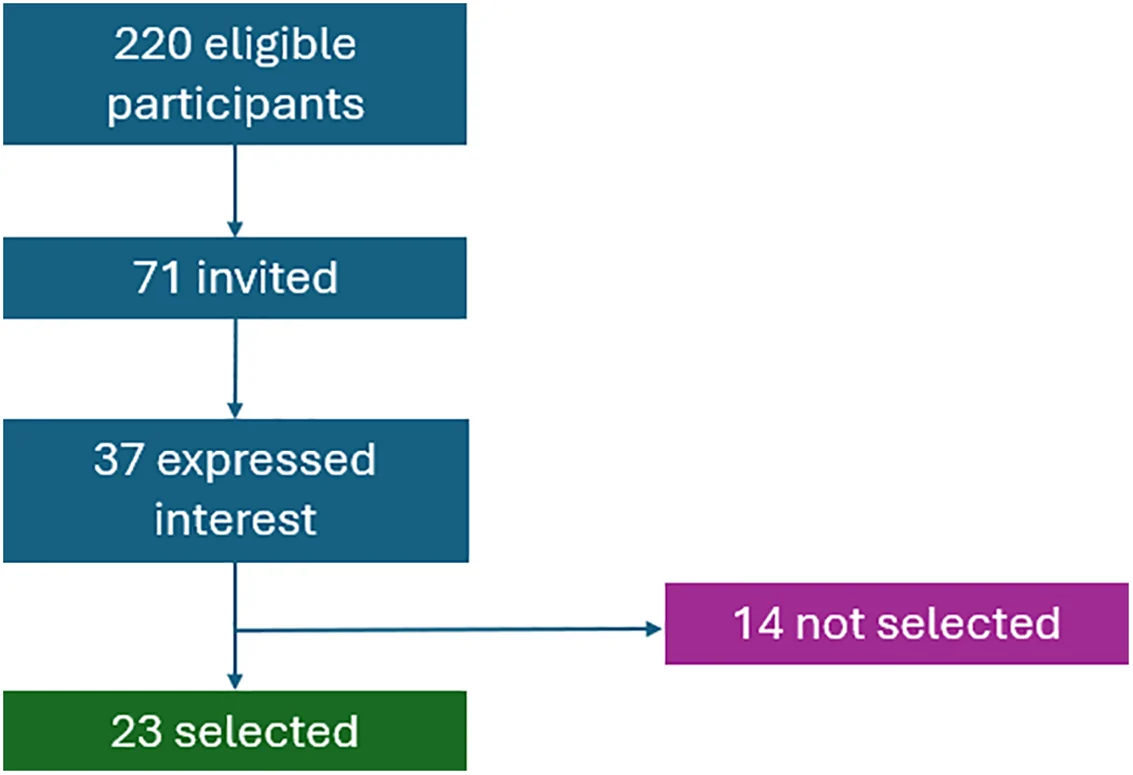
Participant selection.

Between 1^st^ September 2024 and 31^st^ March 2025, 71 of 220 eligible RAMPART participants were invited to join the qualitative sub-study. Recruitment closed early due to a high response rate. 37 participants expressed interest and 23 were purposively selected according to the predefined sampling framework (S3 File; Fig 2).

Median age was 57 years (range 41–76); 14 participants were male and 9 female. All identified as White. 12 were in Arm C and 11 in Arm B; four had experienced recurrence. Characteristics are presented in Table 1.

**Table 1:** Participant characteristics.

		Number (%) n = 23
Age at randomisation	Median (range)	57 (41-76)
Age categories	≤44	4 (17)
	45-59	10 (43)
	60-74	7 (30)
	≥75	2 (9)
Gender	Male	14 (61)
	Female	9 (39)
Ethnicity	White	23 (100)
	Other	0
Toxicity	Any grade	23 (100)
	≥G3	12 (52)
Recurrence	Yes	4 (17)
	No	19 (83)
Arm	Durvalumab	11 (48)
	Durvalumab and Tremelimumab	12 (52)

### Treatment completion and toxicity

Treatment completion and toxicity data were obtained from the RAMPART dataset. All participants had completed trial treatment by the time of interview. 10 received all planned treatment; 13 (57%) discontinued early (eight in Arm C; five in Arm B) due to toxicity (n = 10), COVID-19 disruption (n = 2), or patient choice (n = 1).

All participants reported at least one AE, 12 (52%) had grade ≥3 toxicity (Table 1). Common grade ≥3 AEs included laboratory abnormalities (9/23, 39%), gastrointestinal (5/23, 22%), and endocrine (2/23, 9%). Median AE duration was 29 days (IQR 8–118), and 15 participants (65%) had unresolved AEs at data cut-off. Nine participants (39%) received corticosteroids; one required additional immunosuppressive therapy for immune-mediated myasthenia gravis. Full AE frequencies, grading and duration are provided in the S5 and S6 File.

### Interview procedures

23 interviews were conducted between September 2024 and April 2025 (14 online, 7 telephone, 2 in person). Median time from treatment completion to interview was 33.4 months (range: 23.4–66.5). Median interview duration was 40 minutes (range 25–78).

### Interview findings

**Fig 3:**
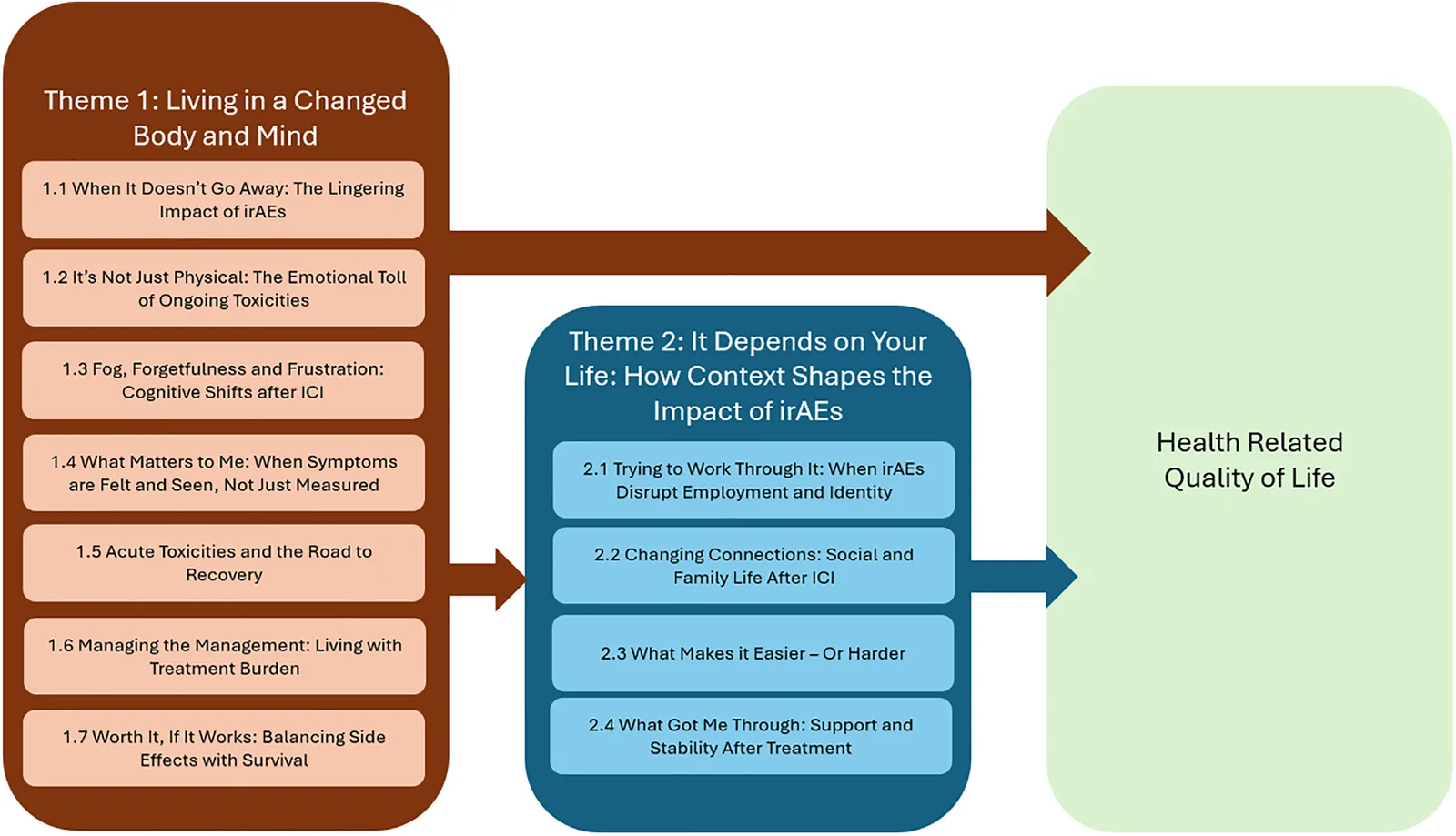
Overall thematic framework: how irAEs affect health-related quality of life.

Two themes were generated from the transcript analysis: *‘Living in a Changed Body and Mind’* and *‘It Depends on Your Life: How Context Shapes the Impact of irAEs’*. Each theme comprises several subthemes (Fig 3). Quotes are anonymised using unique identifiers.

### Theme 1: Living in a changed body and mind

Participants described a range of physical and psychological changes during and after ICI treatment. They also reflected on hypothetical scenarios, which were used to explore how the duration of irAEs, rather than their clinical severity, might influence HRQoL. Across both lived experience and scenario responses, duration emerged as the most important factor shaping impact.

#### Subtheme 1.1 When it does not go away: the lingering impact of irAEs.

For some, persistent symptoms long after treatment stopped created ongoing disruption and uncertainty. Even low-grade irAEs interfered with daily functioning and wellbeing.

*“The side effects that I've had to deal with have gone on some of them for a very long time. And it's not a case of they lessen - it's a case of you work out how you can work around them.”* (P5)

Fatigue was the most common and debilitating chronic symptom, often described as “bone deep” and unpredictable, with a cumulative toll on functioning.

*“It’s not just tiredness, it’s like your body just gives up. You can’t push through it.”* (P7)

Arthralgia also persisted for several participants, affecting mobility and comfort over extended periods. While some experienced intermittent discomfort, others described widespread, ongoing pain that interfered with daily activities.

*“It started in my knees, then my wrists… it’s like my whole body was aching.”* (P11)

Endocrine irAEs were common chronic toxicities. Hypothyroidism was generally manageable and minimally affected HRQoL. In contrast, adrenal insufficiency was complex and burdensome, requiring lifelong steroid replacement and strict dosing schedules, creating ongoing anxiety about missed medication or illness.

*“It’s not just a tablet — it’s something I have to think about every day, and if I get it wrong, it’s dangerous.”* (P10)

For some participants, irAEs resulted in a loss of independence and significant lifestyle adjustments. Unpredictable episodes of immobility or the need to remain close to a toilet disrupted routines, eroded confidence, and made planning difficult. Some expressed a sense of being controlled by their illness.

*“Everything I could do before I couldn’t do it. It was horrendous.”* (P22)Exercise and physical activity were particularly important to several participants, contributing to their sense of wellbeing and identity. The inability to engage in these activities had a notable emotional impact.

*“The gym has always been an important thing for me... for me not to manage to get it, that was very hard.”* (P7)

While some participants described significant disruption due to persistent symptoms, others were able to maintain independence and continue with daily routines during and after ICI treatment, particularly when irAEs were mild, short in duration, or asymptomatic.

*“I’ve been able to carry on and do all the things I’ve been doing before.”* (P8)

#### Subtheme 1.2 It’s not just physical: the emotional toll of ongoing toxicities.

For participants with chronic symptomatic toxicities, the emotional burden was profound. Persistent, unpredictable symptoms often led to anxiety, low mood, and in some cases severe depression and suicidal thoughts.

*“I did go through a very dark time where I was contemplating suicide to be honest because I was just fed up of being a burden.”* (P13)

Permanent toxicities such as adrenal insufficiency had particularly significant mental health impacts, linked to both chronic illness and the physiological effects of low cortisol.

*“If you wake up with low cortisol you can feel quite low and depressed.”* (P12)

Others described a gradual emotional toll from persistent symptoms such as fatigue, arthralgia, or diarrhoea, which disrupted daily life and eroded confidence. Fatigue, in particular, was described as emotionally draining, limiting social engagement and contributing to isolation and low mood.

*“Anything like that is incredibly energy sapping. It ruins your social life... the knock-on effect is mentally.”* (P5)

For some, bowel-related symptoms such as faecal urgency and incontinence were particularly distressing, leading to embarrassment, anxiety, and fear of leaving the house.

*“Our life revolves around me going to the toilet in the morning... I was in [a shop] once and I had an accident there… it just drains you of any confidence and it makes you depressed actually. So yeah, I'm frightened of being away from a toilet.”* (P22)

#### Subtheme 1.3 Fog, forgetfulness, and frustration: cognitive shifts after ICI.

Almost half of participants reported cognitive changes during or after ICI, most often memory decline and difficulty concentrating. For many, these symptoms persisted beyond treatment and affected independence, confidence, and daily functioning.

*“I used to have an incredible memory… now I can’t remember what I did yesterday.”* (P23)

Short-term memory deterioration was the most frequent issue, with participants describing forgetfulness, reliance on reminders, and unsettling changes in their usual abilities.

*“It’s as though somebody’s sort of taken a fork and stabbed holes in your memory.”* (P5)

Concentration difficulties were also common, making tasks such as reading or following conversations harder. Even passive activities like watching television became challenging.

*“I feel as though my focus and powers of concentration probably have never been the same as what they were”* (P19)

Attributing cognitive changes solely to ICI was difficult. Participants acknowledged possible roles of ageing, stress, and cancer experience.

*“There’s a debate, is it the trauma I’ve been through or is it the drugs?”* (P23)

#### Subtheme 1.4 What matters to me: when symptoms are felt and seen, not just measured.

Participants distinguished between irAEs they felt and those detected only through blood tests. Asymptomatic toxicities, such as hepatotoxicity, were not seen as affecting HRQoL.

*“I had that problem with my liver, but it didn't affect me physically”* (P3)

Rashes were generally less impactful unless pruritus disrupted sleep or comfort. Visibility and location sometimes affected confidence and social behaviour.

*“If it was a rash on your face... it might make you feel awkward about going out.”* (P6)

#### Subtheme 1.5 Acute toxicities and the road to recovery.

While chronic symptoms were often more disruptive over time, some participants experienced acute, high-grade irAEs that were physically and emotionally traumatic. Episodes such as colitis, pneumonitis, and myasthenic syndrome required hospitalisation and were described as frightening.

*“I did actually think I might actually die. I really couldn’t breathe.”* (P6)

Recovery trajectory shaped how participants evaluated impact. Improvement and reassurance that treatment was working helped them cope.

*“It was nice to feel that I was getting better quicker.”* (P2)

#### Subtheme 1.6 Managing the management: living with treatment burden.

Managing irAEs often required layered interventions, from topical therapies to systemic treatments such as corticosteroids, insulin, hormone replacement, and, in one case, complex immunosuppression. Experiences varied: some found treatments manageable, others reported side effects that compounded burden.

*“The steroids were OK. I didn’t have too many problems.”* (P6)*“I could literally eat for England… I was just continuously starving.”* (P13)

Hormone replacement therapy was generally well tolerated, though some participants expressed a dislike for long-term dependence. Some described a lack of clarity around the transition of care for chronic irAEs such as hypothyroidism, particularly regarding whether responsibility lay with oncology, primary care, or another specialist. This ambiguity was a source of frustration.

*“It didn't really bother me. It was just more inconvenient like tablets.”* (P14)

*“I kept having to ask questions like so who do I get this levothyroxine from?”* (P4)

#### Subtheme 1.7 Worth It, If It works: balancing side effects with survival.

Despite the burden of irAEs, some participants expressed a clear sense of acceptance when they perceived treatment to be effective in preventing reducing the risk of cancer returning. For these individuals, the presence of irAEs was weighed against the value of survival and long-term remission.

*“We are talking about quality of life… but I’m still here, four years after cancer.”* (P8)

### Theme 2: It depends on your life: how context shapes the impact of irAEs

Participants emphasised that the impact of irAEs extended beyond clinical severity, shaped by personal circumstances, social roles, and available support. Employment, financial security, and family dynamics influenced adaptation to treatment-related challenges. These experiences demonstrate that toxicity is not experienced in isolation but in the context of the life it affects.

“*It’s not really the symptom. It’s the individual person and how it affects their life based on what they actually do.”* (P6)

Across Theme 2, participants described contextual factors that shaped the impact of irAEs, including exacerbating factors such as manual work, caregiving responsibilities and unclear care pathways, and protective factors such as flexible routines, supportive relationships, and structured follow-up (Table 2).

**Table 2:** Contextual factors influencing the impact of irAEs.

Exacerbating factors	Domain	Protective factors
Manual Occupation	Work, income & routine	Flexible work schedule
Self-employed		Supportive employer
Single-income household		Retired
Unclear pathways	Clinical care and system navigation	Supportive care teams
		Structured follow-up
	Personal coping and wellbeing	Lifestyle changes (e.g., exercise, stopping drinking)
		Professional counselling
Caring responsibilities	Relational & social support	Support from family

#### Subtheme 2.1 Trying to work through it: when irAEs disrupt employment and identity.

irAEs often disrupted participants’ ability to work, particularly in physically demanding or self-employed roles. Even low-grade symptoms such as fatigue, arthralgia, and diarrhoea were highly disruptive, especially when unpredictable or persistent.

*“Basically I just couldn’t do my job... I wasn't able to walk.”* (P13)

Seemingly minor symptoms, such as dry mouth and dry eyes, also interfered with job tasks. One participant described how these affected her ability to speak on the phone and use a computer, requiring frequent breaks and symptom management throughout the workday.

*“I was using the drops and yeah, just taking regular breaks from the screen.”* (P16)

Reduced work capacity carried an emotional toll. Employment was closely tied to identity and routine, and participants described frustration and loss.

*“I was always very good at what I did… now I don’t do well at work anymore.”* (P12)

Some returned to work prematurely, driven by financial necessity or a desire for normalcy.

*“You feel that you've got to go back to work anyway early... even though I knew... the joint pain was so bad.”* (P7)

In contrast, those with flexible arrangements or supportive employers reported fewer disruptions.

*“I would be able to kind of not be on all the time… I could take things slowly.”* (P20)

For many, illness or frequent appointments led to loss of work, which in turn resulted in loss of earnings. For self-employed or manual workers, even a single day off could result in lost income.

*“If you don’t work, you don’t get paid.”* (P10)

This pressure was particularly acute for participants in single-income households, especially those with caring responsibilities for children.

*“So it’s on me and everything's always been on me. So I put a lot of it down to maybe a kind of stress in that as well.”* (P7)

Some relied family support to manage financially.

*“Yeah, I did have to take more time off than I got paid for, so my family helped out with them, covering costs.”* (P16)In contrast, participants who were retired, had supportive employers, or could work flexibly reported minimal financial disruption. One participant described reassurance from their employer:

*“Do not worry about money, do not worry about your job... regardless of how long it takes.”* (P5)

#### Subtheme 2.2 Changing connections: social and family life after ICI.

Social consequences of ICI treatment varied. Many reported minimal disruption, while others described withdrawal from social situations due to fatigue, anxiety, or reduced confidence. Changes in intimacy and relational roles were also noted.

*“I think it's changed me as a person… I try to avoid social situations sometimes because it's too stressful for me.”* (P12)

*“After the immunotherapy the sex life was non-existent.”* (P13)

Caregiving roles often shifted within households, creating emotional strain. Parenting was generally maintained, though participants expressed guilt over reduced energy and availability.

*“She was doing all the nursing care… I think it really got her down as well.”* (P22)

*“I felt a little bit bad because I wasn't spending as much time, especially with my daughter.”* (P20)

Financial impact extended to lifestyle restrictions for some, such as being unable to afford holidays abroad due to high insurance costs. Others noted that limited finances affected everyday activities and mental wellbeing.

*“I cannot go abroad anymore now... Where we should be enjoying it we can't afford it. We can't do it now. So, the insurance costs as much as the holiday itself.”* (P22)

#### Subtheme 2.3 What makes it easier – or harder.

The impact of irAEs on daily life was shaped not only by their nature and duration but also by individual circumstances. Employment status and work context, baseline health and daily routines influenced how manageable symptoms felt. Geography added another layer, while routine appointments were generally manageable, long travel times amplified fatigue and emotional strain.

*“Transport is really difficult for me because I have to get a train and that took an hour and a half each way.”* (P12)

Out-of-pocket costs were another consideration. While not universal, some participants highlighted costs of travel and parking.

*“It would have cost me about £150 to park [for treatment for the year] ... which is a consideration, isn’t it for people?”* (P4)

#### Subtheme 2.4 What got me through: support and stability after treatment.

Some participants sought professional support to manage the psychological impact of treatment. Counselling provided a valuable outlet for processing the emotional toll of chronic symptoms and the broader cancer experience.

*“I did go to a counsellor once. And he was so brilliant… He opened my floodgates and I let it all out.”* (P14)

Emotional responses varied. Some reported resilience or even improvement, often linked to lifestyle changes, strong support systems, or a renewed sense of purpose through trial participation.

*“I actually felt better mentally because I wasn’t drinking anymore, and I had something to focus on.”* (P03)

Support from family and clinical teams played a crucial role in coping with treatment demands and anxiety.

*“My wife is perfect. You know, she's so understanding. She's been my rock. If it wasn't for her, then I probably wouldn't be here.”* (P13)

For some, treatment itself became a psychological safety net. Its end brought new challenges, including anxiety about recurrence and vulnerability without regular oversight.*“I missed my visits there… uncertainty, I guess, about whether stopping it meant the likelihood of it returning had increased somewhat.”* (P19)

### Recommendations

Findings informed a set of recommendations for trial design, clinical care, psychological support, practical assistance, and future research. Key areas included improving reporting of irAE duration and need for ongoing management, developing ICI-specific PROMs, clarifying responsibility for long-term toxicity care, integrating psychological support, addressing practical burdens such as travel and financial strain, and exploring cognitive and sexual health impacts (see Table 3).

**Table 3:** Co-developed recommendations.

	Key Finding	Recommendation
**Clinical Trial Design and Reporting**	Even mild or overlapping irAEs, particularly those requiring prolonged steroid use, significantly impact HRQoL.	Report all adverse events, including their duration, management, and whether lifelong treatment is required. Collect data through patient-reported outcome measures (PROMs) and follow-up questionnaires on concomitant medications and late effects.
	Clinicians often prioritise laboratory or clinically serious toxicities, while less clinically serious toxicities such as fatigue are frequently under-recognised. These symptoms can significantly impair HRQoL, particularly when long-lasting.	Use PROMs to capture the patient experience. Report PROMs promptly using standardised guidelines to inform decision-making. PROMs should include questions on residual irAEs post-treatment and whether pharmacological treatment for irAEs is ongoing.
	Some irAEs, including arthralgia, were not captured in current PROMs, but had a significant impact on HRQoL.	Develop tailored PROMs to capture ICI-related toxicities such as arthralgia. Use tools like WISP (write in three symptoms) to identify additional or rare irAEs that are important to patients.
	Certain PROM domains, such as physical and role function, were more sensitive to detecting change in HRQoL than global scores.	Report specific domains like physical and role function, rather than relying solely on global QoL scores.
**Clinical Care, Symptom Management and Communication**	Persistent or overlapping side effects and long-term steroid use were stressful for patients.	Provide clear information on expected duration and management of side effects, including the possibility of lifelong treatment.
	Inability to exercise negatively affected physical and mental well-being. Patients described exercise as important for maintaining mood and resilience during treatment.	Offer tailored exercise and rehabilitation programs to support physical recovery and promote psychological well-being.
	Lack of clarity regarding responsibility for long-term toxicity management post-treatment caused frustration.	Define responsibility for ongoing care and ensure communication between oncology teams, primary care, and relevant specialities. Provide patients with a written follow-up plan and designated contact for ongoing concerns (e.g., a “care passport”).
**Psychological Support**	Chronic toxicities adversely affected mental health, causing anxiety and low mood.	Offer timely psychological support and ensure patients have a clearly identified contact person or service for mental health concerns. Communicate this information proactively during and after treatment.
	Treatment completion triggered anxiety about recurrence and reduced clinical contact.	Provide structured emotional support post-treatment, such as peer groups or counselling.
**Practical Support: Travel, Work, and Financial Burden**	Physical irAEs limited ability to work, particularly in physically demanding roles. Financial impact was greater for self-employed patients.	Offer early advice on financial support and assistance with travel-related costs (e.g., parking, fuel). Consider signposting to resources for those at higher risk of financial hardship, such as self-employed individuals.
	Travelling to hospital was expensive and tiring, especially for those living far away.	Offer remote appointments, evening/weekend appointments or home care when possible, to reduce travel and time off work.
**Future Research**	Some people felt irAEs were acceptable if treatment was effective.	Investigate the level of toxicity that patients consider acceptable in exchange for improved survival outcomes.
	Cognitive changes were commonly reported. These affected confidence and independence. Sexual function was mentioned by one participant, although this was not specifically explored during interviews.	Conduct research to better understand cognitive effects and explore sexual health concerns. Incorporate screening for cognitive impairment and questions on sexual function into future clinical trials.

## Discussion

Our findings suggest that chronic or unresolved toxicities are more disruptive to daily life than clinician-reported severity grades indicate. Even mild or overlapping irAEs, particularly those requiring prolonged steroid use, disrupted daily functioning and emotional wellbeing. Fatigue was the most burdensome symptom, followed by diarrhoea and arthralgia, while cognitive changes undermined confidence and autonomy. Physical limitations, including reduced ability to exercise, negatively affected wellbeing. The impact of irAEs was context dependent. Participants in physically demanding or self-employed roles experienced greater disruption than those who were retired, and financial stress was amplified in single-income households. Some viewed irAEs as acceptable when treatment was effective, others expressed frustration about chronic symptoms and lack of coordinated care.

ICIs are now recognised to cause chronic, delayed and sometimes permanent irAEs that may require lifelong management [11–13,15]. However, clinical trials rarely report the duration or resolution of irAEs or the need for ongoing immunosuppressive or hormone replacement therapy, information which is critical for risk–benefit assessment and patient counselling. Historically, inconsistent terminology has resulted in variability in reporting and difficulties comparing chronic toxicity across studies. Recent consensus definitions from the Society for Immunotherapy of Cancer (SITC) provide a framework for standardised reporting, distinguishing late irAEs (occurring after treatment cessation) and chronic irAEs (persisting beyond 12 weeks) [29]. Despite this, most registration trials base safety analyses on short follow-up periods, and a systematic review found that 90% did not specify the proportion of patients with ongoing toxicity at data cut-off [30]. Conventional safety reporting may therefore underestimate the incidence and persistence of chronic irAEs.

Evidence on the impact of ICIs on HRQoL remains mixed. Randomised controlled trials frequently report no clinically meaningful differences in global HRQoL, despite high rates of grade 3/4 irAEs and treatment discontinuation in some trials [31–33]. In contrast, observational and qualitative studies highlight a broader range of physical, cognitive, psychological, and social challenges, as well as financial toxicity, consistent with our findings [33,34]. A growing body of studies have examined survivorship after ICIs, but few have explored how chronicity of irAEs impacts HRQoL. Schulz et al. conducted a multicentre cross-sectional study of 200 ICI-treated patients (≥12 weeks post-treatment) compared with individuals with non-ICI-induced autoimmune disease [35]. Chronic irAEs were associated with significantly reduced HRQoL compared to ICI-treated patients without chronic irAEs and to normative population values, with impairment comparable to autoimmune disease. The study included mixed cancer types and stages but did not specify the proportion with early-stage disease, which is an important consideration because chronic toxicity may be less acceptable to patients who may already be cured of cancer.

Several systematic reviews highlight that existing PROMs lack content validity in ICI-treated populations [19,21,22]. The absence of observed differences in HRQoL may reflect limitations in measurement rather than true equivalence; without appropriate instruments, clinically meaningful effects are likely to remain undetected. This underscores limitations of generic tools like EORTC QLQ-C30 and the need for ICI-specific PROMs. Development work is underway: the EORTC has produced an ICI-specific module (IL453), with further validation planned, and RCC-specific tools are also in progress, including the EORTC RCC module, currently in preliminary testing, and the FKSI-23, which is undergoing validation [36–38]. Another challenge is reporting global QoL rather than domain and symptom scales. For example, one case-control study comparing long-term advanced melanoma survivors treated with anti-CTLA-4 ICIs showed worse functioning scores and more symptom burden, but no difference in global QoL [39]. This may reflect response shift in global scores, where patients recalibrate expectations after a life-threatening diagnosis [40,41]. Sole reliance on mean HRQoL scores can also obscure clinically significant changes in subsets of patients. For example, a recent real-world study of patients with resected melanoma treated with adjuvant anti-PD-1 therapy found mean symptom and functioning scores improved or remained stable at 12 months, yet a substantial proportion experienced clinically significant declines in role, social or emotional functioning, particularly younger patients [42].

Participants in our study who developed chronic or permanent toxicities described a range of emotional impacts, including anxiety and depression. These were often linked to persistent symptoms that disrupted daily routines, work ability and financial security, and created uncertainty about recovery. Some participants reported diminished confidence and autonomy. These observations align with other studies showing high levels of anxiety and depression among ICI survivors [34,35,43–45]. In our study one participant with adrenal insufficiency described episodes of low mood attributed to cortisol deficiency and anxiety about managing steroid replacement, illustrating the complex interplay between endocrine irAEs and psychological wellbeing.

Our findings of high levels of perceived cognitive impairment align with reports that ICIs may affect neurocognitive function [39,43,44,46–48]. Objective testing in ICI treated melanoma cohorts has shown impairment in up to 41% of survivors, often persisting beyond treatment [43,44]. However, prior studies note poor correlation between subjective complaints and objective performance, suggesting that perceived deficits may reflect broader psychosocial factors [46]. Previous research has described memory and concentration problems impacting daily activities such as work, household tasks, and leisure, patterns mirrored in our cohort [47]. Unlike studies incorporating objective testing, we assessed cognition subjectively, but our results reinforce the need for further research.

Although trial participation mitigated some costs, several participants reported financial strain related to time off work, travel, and out-of-pocket expenses. This echoes real-world studies where financial toxicity is a recognised burden and correlates with a lower HRQoL [35, 49]. In our cohort, financial strain was most common among participants with caring responsibilities or those in manual or self-employed roles who lost income because of missed workdays.

### Strengths and limitations

This study included a diverse sample in age, gender, employment, caregiving status, and experiences of toxicity and recurrence, enhancing the breadth of perspectives. Flexible recruitment and multiple interview modalities improved accessibility. In-depth interviews generated rich data, and analysis was strengthened by multidisciplinary review and co-development of the thematic framework with clinical and non-clinical researchers. Importantly, recommendations were co-developed with patients and refined by an expert panel, ensuring they reflect patient priorities while being clinically relevant, feasible and practical.

Limitations included the exclusively UK-based cohort, which may mean experiences such as financial toxicity were less prominent than in other healthcare settings. The sample was drawn exclusively from the RAMPART trial and, despite targeted recruitment, lacked ethnic diversity, with all participants identifying as White. Ethnic minority representation within the eligible population was also limited, with only six (3%) of the 220 eligible participants identifying as Asian (n = 5) or Black (n = 1). As experiences of treatment and its impact on daily life may differ across ethnic groups and cultural contexts, transferability of these findings to more ethnically diverse populations should be considered with caution. The sample was purposively selected to capture a breadth of toxicity experiences (including more impactful or higher‑grade irAEs) to explore HRQoL effects; it was not intended to reflect toxicity incidence within RAMPART or the wider ICI‑treated population. We recognise that non-participants may have held different perspectives that are not captured in the present study and that transferability of the findings should be considered in this context.

Not all irAEs were represented, though participants consistently emphasised that the impact of irAEs on daily life, rather than the specific event, shaped their experiences. Sexual health and fertility concerns were not systematically assessed and were rarely raised, which may reflect the interview guide and cohort characteristics. These domains remain underexplored in ICI survivorship, despite evidence that sexual function issues affect a substantial proportion of patients [34,48]. This study was not designed to compare experiences of participants who received ICIs with those who did not, so we cannot determine whether some reported issues such as fatigue or psychological concerns are specific to ICI treatment or reflect broader experiences of cancer survivorship. The recall period was long (median 33.4 months) which may have limited recall accuracy; however, this enabled exploration of chronic toxicities and survivorship issues. Although steps were taken to mitigate power dynamics, there was still potential bias as SM is an oncology clinician. Interviews were conducted in neutral settings, participants were not known to SM, and none were treated at hospitals where she worked, but some may have perceived her as linked to the RAMPART trial and felt reluctant to express negative views. A potential strength of SM’s clinical background was the ability to explore the impact of certain irAEs in greater depth.

### Recommendations

Our findings highlight the need for systematic collection and reporting of irAE data including duration, resolution and the need for ongoing management as these issues significantly impact patients’ HRQoL. Validated ICI-specific PROMs should be used to assess HRQoL, with domain-level reporting rather than relying solely on global QoL scores, which risks masking clinically significant changes.

In clinical practice, persistent toxicities require clear communication about expected duration, management strategies, and responsibility for ongoing care. Structured follow-up plans and designated contacts could reduce uncertainty and improve continuity. Management should be coordinated between oncology, primary care, and relevant specialty teams. Tailored exercise and rehabilitation programs should support physical recovery and psychological resilience.

Psychological support must be integrated into survivorship pathways, with proactive communication about available services and structured emotional support post-treatment. Practical measures, such as remote consultations, flexible scheduling, and early financial advice, can mitigate travel and work-related burdens, particularly for self-employed patients.

Finally, future research should explore cognitive and sexual health impacts of treatment and define what level of toxicity patients consider acceptable in exchange for improved survival outcomes.

## Conclusion

Our findings suggest that chronic immune-related toxicities negatively impact HRQoL, with wide-ranging physical, cognitive, psychological, and social consequences. Addressing these challenges requires systematic irAE reporting in trials, validated ICI-specific PROMs with domain-level analysis, and integrated survivorship care that spans oncology, primary care, and specialist teams. Structured follow-up, tailored rehabilitation, psychological support, and practical assistance are essential to improve outcomes for patients living with persistent treatment effects.

## Supporting information

S1 File COREQ checklist. (DOCX)

S2 File Interview guide. (DOCX)

S3 File Purposive sampling frame. (DOCX)

S4 File Final thematic framework. (DOCX)

S5 File Frequency and severity of adverse events. (DOCX)

S6 File Duration of adverse events by grade. (DOCX)
