# Supplementary material for: Health-related quality of life with adjuvant immune checkpoint inhibition in renal cell carcinoma: A qualitative study of UK participants in the phase III RAMPART trial
Source: PLoS One. 2026 Sep 21;21(9):e0358789. doi: 10.1371/journal.pone.0358789 (PMC13592578; doi:10.1371/journal.pone.0358789)
Supplement: Supplementary file 1 [file pone.0358789.s001.docx]

# **Supporting information (S1)**

## **Consolidated criteria for reporting qualitative studies (COREQ): 32-item checklist**

| Domain/Item | Description | How Addressed in Manuscript |
| --- | --- | --- |
| Domain 1: Research team and reflexivity |  |  |
| Personal characteristics |  |  |
| 1. Interviewer/facilitator | Which author conducted interviews? | SM conducted all interviews (Methods: Data Collection). |
| 2. Credentials | What were the researcher’s credentials? | SM is an oncology clinician with prior experience of qualitative research (Methods: Study Design and Research Team). |
| 3. Occupation | What was their occupation at the time of the study? | SM is an oncology clinician and clinical research fellow (Methods: Study Design and Research Team). |
| 4. Gender | Was the researcher male or female? | SM is a female clinical research fellow (Methods: Study Design and Research Team). |
| 5. Experience and training | What experience or training did the researcher have? | SM and team had prior qualitative research experience (Methods: Study Design and Research Team). |
| Relationship with participants |  |  |
| 6. Relationship established | Was a relationship established prior to study commencement? | No participants known to SM or treated at a hospital where she worked (Methods: Participants and Recruitment). |
| 7. Participant knowledge of the interviewer | What did the participants know about the researcher? | Participants informed of interviewer’s role as oncology clinician and researcher (Methods: Data Collection). |
| 8. Interviewer characteristics | What characteristics were reported about the interviewer/facilitator? | SM was a female oncology clinician and clinical research fellow (Methods: Study Design and Research Team). |
| Domain 2: Study design |  |  |
| Theoretical framework |  |  |
| 9. Methodological orientation and Theory | What methodological orientation was stated to underpin the study? | Study conducted within critical realistic paradigm (Methods: Study Design and Research Team). |
| Participant selection |  |  |
| 10. Sampling | How were participants selected? | Sampling was purposive, aiming for diversity in age, gender, ethnicity, trial arm and toxicity experience (Methods: Participants and Recruitment). |
| 11. Method of approach | How were participants approached? | Invited via trial sites after central identification (Methods: Participants and Recruitment). |
| 12. Sample size | How many participants were in the study? | 23 interviews (Results: Participant Recruitment). |
| 13. Non-participation | How many people refused to participate or dropped out? | 71 invited; 23 interviewed (Results: Participant Recruitment). |
| 14. Setting of data collection | Where was the data collected? | Online, phone, or in person (Methods: Data Collection). |
| 15. Presence of non participants | Was anyone else present besides the participants and researchers? | No (Methods: Data Collection). |
| 16. Description of sample | What are the important characteristics of the sample? | Age, gender, trial arm, toxicity experience, recurrence status (Results: Table 1). |
| Data collection |  |  |
| 17. Interview guide | Were questions, prompts, guides provided by the authors? Was it pilot tested? | Yes and it was pilot tested with a patient (Methods: Study Design and Research Team) full guide available in Supporting Information. |
| 18. Repeat interviews | Were repeat interviews carried out? | No (Methods: Data Collection). |
| 19. Audio-visual recording | Did the research use audio or visual recording to collect the data? | Yes, audio+/-visually-recorded (Methods: Data Collection). |
| 20. Field notes | Were field notes made during and/or after the interview or focus group | Yes (Methods: Data Collection). |
| 21. Duration | What was the duration of the interviews or focus group? | Median interview duration was 40 minutes (Results: Interview Procedures). |
| 22. Data saturation | Was data saturation discussed? | Recruitment closed when sampling matrix categories were filled or unfillable (Results: Recruitment). |
| 23. Transcripts returned | Were transcripts returned to participants for comment and/or correction? | No (Methods: Data Collection). |
| Domain 3: Analysis and findings |  |  |
| 24. Number of data coders | How many data coders coded the data? | SM and AS coded; others reviewed excerpts (Methods: Data Analysis). |
| 25. Description of the coding tree | Did authors provide a description of the coding tree? | Yes, thematic framework described; full framework in Supplementary Material (Methods: Data Analysis). |
| 26. Derivation of themes | Were themes identified in advance or derived from the data? | Iterative framework method (Methods: Data Analysis). |
| 27. Software | What software, if applicable, was used to manage the data? | NVivo 14 (Methods: Data Analysis). |
| 28. Participant checking | Did participants provide feedback on the findings? | Final thematic structure reviewed with 10 participants (Methods: Data Analysis). |
| Reporting |  |  |
| 29. Quotations presented | Were participant quotations presented to illustrate the themes / findings? | Yes, anonymised quotes throughout Results section. |
| 30. Data and findings consistent | Was there consistency between the data presented and the findings? | Yes, quotes illustrate themes (Findings). |
| 31. Clarity of major themes | Were major themes clearly presented in the findings? | Yes, two main themes with subthemes (Findings). |
| 32. Clarity of minor themes | Is there a description of diverse cases or discussion of minor themes? | Yes, subthemes detailed under each theme (Findings). |
