# Supplementary material for: Health-related quality of life with adjuvant immune checkpoint inhibition in renal cell carcinoma: A qualitative study of UK participants in the phase III RAMPART trial
Source: PLoS One. 2026 Sep 21;21(9):e0358789. doi: 10.1371/journal.pone.0358789 (PMC13592578; doi:10.1371/journal.pone.0358789)
Supplement: Supplementary file 2 [file pone.0358789.s002.docx]

# **Supporting information (S2)**

## **Interview guide**

## Welcome and Introduction

- Introduce yourself as the researcher.
- Explain the aims and objectives of the study and why this is being done in addition to the questionnaires completed as part of RAMPART.
- Explain confidentiality and anonymity, including circumstances where this may need to be broken (e.g., concern about harm to themselves or others).
- Explain the format of the interview (30–90 minutes) and that participants can take a break if needed.
- Discuss recording and the process for transcription and analysis following the interview. Explain the use of anonymised quotations.
- Remind participants about the £25 gift voucher.
- Explain that they do not have to answer any questions they do not want to and can stop the interview at any time. If they wish to withdraw from the study, they can contact us within two weeks of the interview and we will delete all data. Beyond that point, data cannot be deleted as analysis will have begun.
- Check whether they have any questions.
- Confirm they are happy to continue and start recording.

**START RECORDING**

## Definition of health-related quality of life

- Health-related quality of life looks at the impact your health has on your overall quality of life. This considers a combination of:
- **Physical wellbeing** – includes symptoms such as pain or vomiting, and the ability to carry shopping or take a walk outside the house.
- **Mental wellbeing** – for example, feeling worried, irritable, depressed, or having difficulty concentrating.
- **Social wellbeing** – being able to keep up with social activities and the impact on finances.
- Quality of life is a broad term and different people value different things. We would like to know the impact of immunotherapy treatment on *your* quality of life.

## Treatment history, side effects and impact on HRQoL

*Aim: to get patient talking and find out about their personal experience with immunotherapy, the side effects they experienced and whether they felt this impacted their HRQoL.*

- Could you start by telling me about your situation before you were diagnosed with cancer. What was a typical day like for you?

Prompts: physical health/fitness, living situation, mental health, cognition, relationships, work, hobbies/interests

- And then can we talk about your diagnosis of kidney cancer and how you were when you were about to start RAMPART?
- How were you during your treatment?
- **Side effects:** Did you experience any side effects? Were you admitted to hospital? Did you need to delay or stop treatment? Did you need to take steroids or other immunosuppressants? Did you need to adapt your way of life to cope with side effects? If you had chronic or persistent side effects did you adapt to these over time?
- Did you complete all of your treatment?
- How did receiving immunotherapy affect you?

Prompts: physical health/fitness, living situation, mental health, cognition, relationships, work, hobbies/interests, finances

- What is a typical day like for you now?
- Has your cancer recurred?
- Knowing what you do now, and given your experience, would you have immunotherapy again?

## Duration/severity of toxicity and impact on HRQoL

*Aim: to understand importance of duration, severity, and burden of toxicity on HRQoL*

- Show participant 3 individual vignettes (printed in person, slide if virtual, describe if telephone):

**Note:** The specific toxicities (e.g., diarrhoea, breathlessness, rash) and timeframes were varied across interviews to ensure responses were not influenced by a single symptom or fixed duration.

1. **Severity:** Patient receiving 1 year of immunotherapy for localised kidney cancer to prevent cancer coming back. After 3 months of treatment admitted to hospital with diarrhoea (8x per day) and abdominal pain. Diagnosed with inflammation of the bowel because of immunotherapy. Needs to stay in hospital for 2 weeks for intravenous steroids and is then discharged home on tablet steroids which are reduced over the next 8 weeks. This side effect resolves and does not happen again.
2. **Duration:** Patient receiving 1 year of immunotherapy for localised kidney cancer to prevent cancer coming back. Develops diarrhoea 3 times per day three months into treatment. Persists >1 year after finishing treatment. Hospital admission is not required. (DURATION).
3. **Burden:** Patient receiving 1 year of immunotherapy for localised kidney cancer to prevent cancer coming back. 3 months into treatment develops diarrhoea three times/day (G1), fatigue which gets better when they rest (G1), and a rash on the abdomen and chest which is itchy and red (G1). Hospital admission is not required. These symptoms last for three months then resolve.

- For each vignette, ask if they feel this would negatively impact HRQoL
- Then ask them to rank scenarios in order of which would most negatively impact HRQoL and discuss why they have ordered them this way.
- What would they consider to be a severe side effect?

Prompts: high dose steroids, need for hospital admission, impact on ability to carry out usual activities

- If you needed to receive high dose steroids, how long would you consider to be problematic for you?

## Conclusion

- Thank participant for their time.
- Ask if they have anything they would like to add before recording stops.
- Would you be happy for me to keep contact details so that we can invite you to review the findings of this study?
- Provide contact details if they have further questions.

**STOP RECORDING**
