# Supplementary material for: Health-related quality of life with adjuvant immune checkpoint inhibition in renal cell carcinoma: A qualitative study of UK participants in the phase III RAMPART trial
Source: PLoS One. 2026 Sep 21;21(9):e0358789. doi: 10.1371/journal.pone.0358789 (PMC13592578; doi:10.1371/journal.pone.0358789)
Supplement: Supplementary file 3 [file pone.0358789.s003.docx]

# **Supporting information (S3)**

## **Purposive sampling framework**

| **Characteristic** | **Minimum Target** | **Achieved** |
| --- | --- | --- |
| Gender   - Female - Male | 6  6 | 9  14 |
| Age range   - ≤44 - 45-59 - 60-74 - ≥75 | 3  3  3  3 | 4  10  7  2 |
| Ethnicity   - White - Black - Asian | 3  3  3 | 23  0  0 |
| Toxicity   - No - Yes – Grade 1-2 Adverse Events - Yes – Grade 3-4 Adverse Events | 3  3  3 | 0  11  12 |
| Trial Arm   - Durvalumab - Durvalumab plus Tremelimumab | 3  3 | 11  12 |
