# Supplementary material for: Health-related quality of life with adjuvant immune checkpoint inhibition in renal cell carcinoma: A qualitative study of UK participants in the phase III RAMPART trial
Source: PLoS One. 2026 Sep 21;21(9):e0358789. doi: 10.1371/journal.pone.0358789 (PMC13592578; doi:10.1371/journal.pone.0358789)
Supplement: Supplementary file 4 [file pone.0358789.s004.docx]

# **Supporting information (S4)**

## **Final thematic framework**

| Physical Health | | | | Emotional Health | Cognitive Health | Functioning and Daily Life | Financial Toxicity |
| --- | --- | --- | --- | --- | --- | --- | --- |
| Physical Function | Severity of irAEs | Duration of irAEs | Treatment of irAEs |  |  |  |  |
