# Supplementary material for: Health-related quality of life with adjuvant immune checkpoint inhibition in renal cell carcinoma: A qualitative study of UK participants in the phase III RAMPART trial
Source: PLoS One. 2026 Sep 21;21(9):e0358789. doi: 10.1371/journal.pone.0358789 (PMC13592578; doi:10.1371/journal.pone.0358789)
Supplement: Supplementary file 5 [file pone.0358789.s005.docx]

# **Supporting information (S5)**

## **Frequency and severity of adverse events in RAMPART qualitative sub-study participants**

| **System Organ Class** | **All grades**  **n (%)** | **Grade 3**  **n (%)** | **Grade 4**  **n (%)** |
| --- | --- | --- | --- |
| Investigations (laboratory abnormalities) | 22 (96) | 9 (39) | 1 (4) |
| Gastrointestinal disorders | 19 (83) | 5 (22) | 0 |
| Nervous system disorders | 15 (65) | 1 (4) | 0 |
| Skin and subcutaneous tissue disorders | 14 (61) | 2 (9) | 0 |
| Metabolism and nutrition disorders | 12 (52) | 0 | 0 |
| Musculoskeletal and connective tissue disorders | 12 (52) | 0 | 0 |
| Respiratory, thoracic and mediastinal disorders | 12 (52) | 1 (4) | 0 |
| Blood and lymphatic system disorders | 10 (43) | 0 | 0 |
| Infections and infestations | 10 (43) | 0 | 0 |
| General disorders and administration site conditions | 9 (39) | 1 (4) | 0 |
| Psychiatric disorders | 8 (35) | 1 (4) | 0 |
| Eye disorders | 7 (30) | 0 | 0 |
| Renal and urinary disorders | 7 (30) | 1 (4) | 0 |
| Vascular disorders | 6 (26) | 1 (4) | 0 |
| Endocrine disorders | 5 (22) | 2 (9) | 1 (4) |
| Cardiac disorders | 4 (17) | 1 (4) | 0 |
| Injury, poisoning and procedural complications | 2 (9) | 0 | 0 |
| Reproductive system and breast disorders | 1 (4) | 0 | 1 (4) |
| Immune system disorders | 1 (4) | 0 | 0 |
| Neoplasms (benign, malignant and unspecified) | 1 (4) | 0 | 0 |
| Ear and labyrinth disorders | 1 (4) | 0 | 0 |
| Surgical and medical procedures | 1 (4) | 1 (4) | 0 |

*Percentages are based on n=23 participants. Grade refers to CTCAE Version 5. Adverse events are presented according to the Medical Dictionary for Regulatory Affairs system organ class. Numbers represent highest toxicity grade (i.e. each participant is counted only once in each row). No grade 5 adverse events occurred.*
