# Supplementary material for: Health-related quality of life with adjuvant immune checkpoint inhibition in renal cell carcinoma: A qualitative study of UK participants in the phase III RAMPART trial
Source: PLoS One. 2026 Sep 21;21(9):e0358789. doi: 10.1371/journal.pone.0358789 (PMC13592578; doi:10.1371/journal.pone.0358789)
Supplement: Supplementary file 6 [file pone.0358789.s006.docx]

# **Supporting information (S6)**

## **Duration of adverse events by grade for each participant**
